# Successful Surgical Treatment of an Infected Thoracoabdominal Aneurysm Accompanied with Leriche Syndrome

**DOI:** 10.1155/2019/1628157

**Published:** 2019-04-21

**Authors:** Masato Furui, Hirohisa Hirata, Bunpachi Kakii, Gaku Uchino, Mai Asanuma, Haruo Suzuki, Hiroaki Nishioka, Takeshi Yoshida

**Affiliations:** ^1^Cardiovascular Surgery Department, Matsubara Tokushukai Hospital, 7-13-26 Amamihigashi, Matsubara, Osaka, Japan; ^2^Surgery Department, Matsubara Tokushukai Hospital, 7-13-26 Amamihigashi, Matsubara, Osaka, Japan; ^3^Cardiovascular Surgery Department, Kyoto Renaiss Hospital, 4-13, Suehirocho, Fukuchiyama, Kyoto, Japan

## Abstract

A 56-year-old man presenting with massive melena and loss of consciousness was diagnosed with an infected thoracoabdominal aneurysm, an aortoduodenal fistula, and Leriche syndrome following an evaluation by computed tomography. Emergency surgery for uncontrolled infection included the reconstruction of the superior mesenteric and bilateral renal arteries using a four-branched graft. The aortoduodenal fistula was resected after omental filling, and an enterostomy was performed for feeding. Intestinal reconstruction was performed in two stages. The patient was discharged on postoperative day 48 and was without evidence of recurrence at 23 months postoperatively.

## 1. Introduction

Leriche syndrome, or aortoiliac occlusive disease, usually presents with claudication, erectile dysfunction, and absence of a lower limb pulse and is often a result of arteriosclerosis obliterans in the lower limb. The study patient proved to be an extremely rare case of Leriche syndrome accompanied by an infected thoracoabdominal aortic aneurysm [1–5].

## 2. Case Presentation

**Figure 1:**
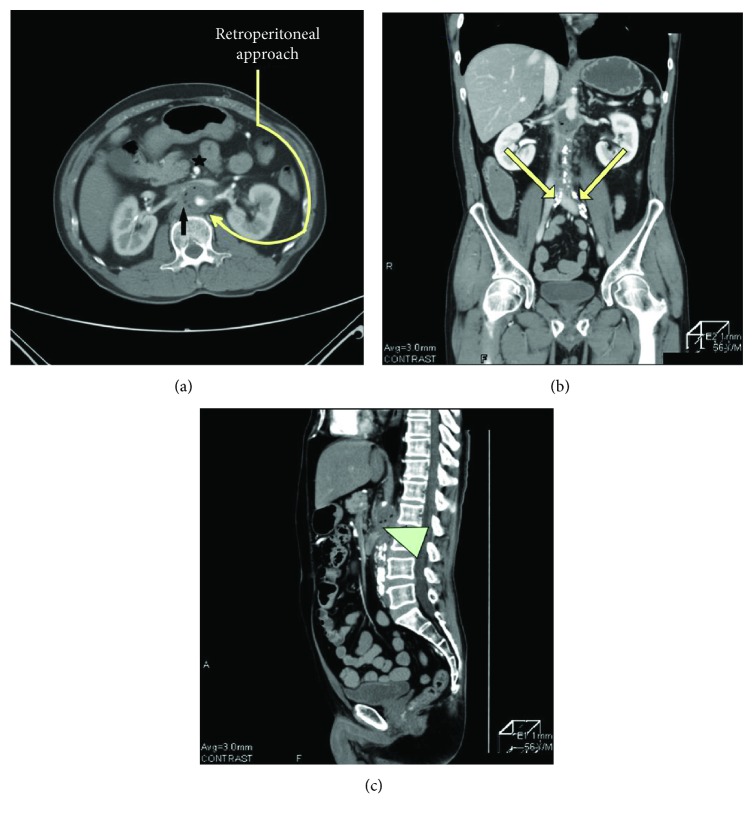
Preoperative enhanced computed tomography indicates an infected aortic aneurysm at the thoracoabdominal level. (a) Small aneurysm with intra-aortic air (arrow), the superior mesenteric artery (star), and renal artery in the axial view. (b) Obstructed distal abdominal aorta and common iliac arteries (arrows) with calcification in the coronal view. (c) Suspected aortoduodenal fistula (arrowhead) in the sagittal view.

A 56-year-old man was transported to our hospital because of massive melena and loss of consciousness. He was a current smoker with a history of hyperlipidemia. Physical examination revealed no inguinal pulse. He complained of mild chronic claudication despite having no impotence, and computed tomography revealed aortoiliac occlusive disease owing to atrophy and calcification of the terminal aorta and iliac arteries. His vital signs included a blood pressure of 135/70 mmHg and pulse of 90 beats/min. The white blood cell count was 16,300 cells/mm^3^, hemoglobin was 10.5 g/dL, and C-reactive protein level was 1.93 mg/L. A 35 mm thoracoabdominal aneurysm with intra-aneurysmal gas was seen on enhanced computed tomography (Figure 1), consistent with an infected thoracoabdominal aneurysm accompanied by Leriche syndrome.

**Figure 2:**
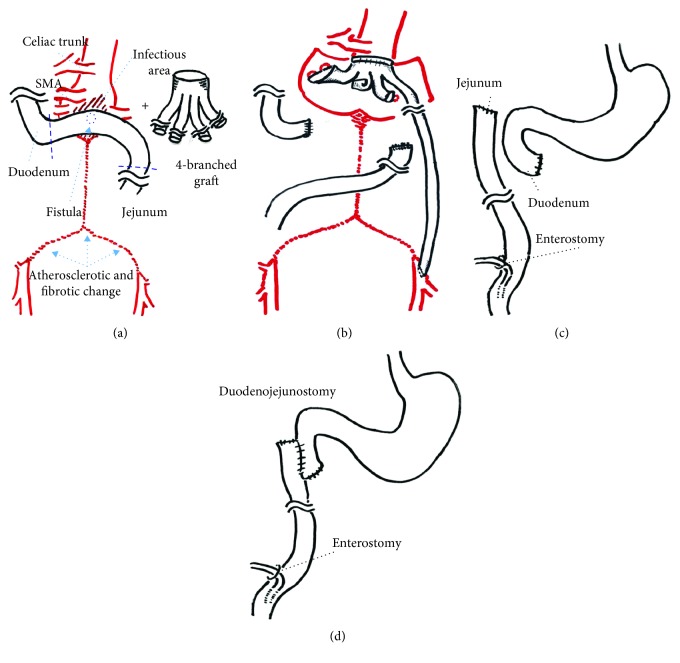
Diagram of the surgical procedure. (a) Aneurysm, aortoduodenal fistula (arrow), and a 4-branched graft are shown. Oblique lines show the infectious area. Wavy lines are resected areas of the duodenum and jejunum. (b) Aortic repair and left leg revascularization are shown. The aortoduodenal fistula was subsequently resected. (c) Enterostomy was attached to the jejunum as the first stage of intestinal reconstruction. (d) The duodenojejunostomy was performed as the second stage of intestinal reconstruction.

**Figure 3:**
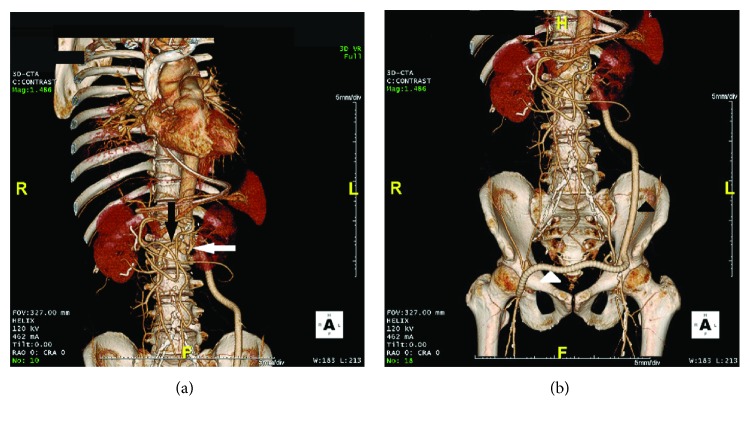
Postoperative three-dimensional reconstructed computed tomography scan shows the graft of the thoracoabdominal aorta and the additional femorofemoral bypass graft. (a) The superior mesenteric artery and right renal artery (black arrow) and the left renal artery (white arrow) were reconstructed with three of four leg grafts. (b) The left common femoral artery was bypassed with the remaining left leg graft (black arrowhead). The right common femoral artery was also bypassed with an 8 mm graft (white arrowhead).

Emergency surgery was performed under general anesthesia with the patient in the right semilateral position. The procedure began with a left thoracoabdominal incision through the seventh intercostal space. When we reached the aorta through careful retroperitoneal approach, we could not reserve the left inferior epigastric artery, running obliquely around the incision area (Figure 1). The color of aortic aneurysm wall changed from yellowish-white to black-brown. The aorta was clamped above the celiac trunk because the infection had spread around the superior mesenteric artery (SMA). Then, the infected aneurysm was opened under partial cardiopulmonary bypass (CPB) via the femoral artery and vein and thoroughly debrided. The abdominal branch vessels were perfused by CPB, and the opposite femoral artery of the sending cannulation was also perfused through a sheath connected to CPB. The celiac trunk appeared normal and was not involved by the aneurysm, and the aorta below the celiac trunk was replaced. Since the aorta had a blind end because of Leriche syndrome, we used a knitted graft with four branches (INTERGARD quarto 18-9 mm; Maquet Getting Group, Tokyo, Japan) (Figure 2(a)). The SMA and right renal artery were reconstructed using the right graft branch, and the left renal artery was reconstructed with the left graft branch (Figures 2(b) and 3(a)). The second left graft branch was passed to the left common femoral artery because of the left lower limb ischemia by sacrificing the inferior epigastric artery. The omentum was filled around the graft and debridement cavity. The procedure time was 497 min and CPB time was 124 min. After the vessel reconstruction, a horizontal segment of the duodenum containing a 5 mm fistula was resected (Figures 2(a) and 2(b)). The ends of the duodenum and proximal jejunum were closed by an intestinal two-stage reconstruction; the first stage includes removal of the aortoduodenal fistula and stump of the edges, and the second stage includes a duodenojejunostomy. And the intestinal fistula was made for feeding (Figure 2(c)). On postoperative day 1, the patient complained of severe pain in the right lower limb on sitting. A Doppler evaluation of the bilateral dorsalis pedis arteries revealed right lower limb ischemia. Femorofemoral bypass was performed using an ePTFE graft (Gore Propaten 8 mm; WL Gore & Associates, Co. Ltd., Tokyo, Japan) attached to the side of the leg graft branch (Figure 3(b)).

*Streptococcus* and *Peptostreptococcus asaccharolyticus* were isolated from the aneurysm. Vancomycin and meropenem were administered for 1 month, and a duodenojejunostomy, as the second stage, was performed on day 30 after the inflammation subsided (Figure 2(d)). Antibiotic was changed to oral levofloxacin on day 43, and the intestinal fistula was removed on day 44. The patient was discharged on day 48 and continued antibiotics at home for 6 months. To the best of our knowledge, there has been no evidence of any complications such as graft infection and pseudoaneurysm for 23 months postoperatively.

This study was approved by the Ethics Committee of Matsubara Tokushukai Hospital (approval number: 171202). The patient consented to the publication of this report.

## 3. Discussion

Only 0.7%–3% of all aneurysms are infected, and those at the thoracoabdominal level are extremely rare [1–4]. The challenges of successful treatment include the revascularization in the infected territory, control of sepsis, and reconstruction of the digestive tract. Thoracic endovascular aortic repair for Leriche syndrome or infected aneurysms has been reported [5, 6]. In this case, open surgery was required because fibrosis and atherosclerosis of the iliac arteries made an endovascular approach impossible and debridement of the infected aorta was desirable. The revascularization of an infected aortic aneurysm by in situ or nonanatomical reconstruction is controversial. Yasuda et al. reported that extra-anatomical bypass was safe and reliable [2], and others have reported successful in situ reconstruction [1, 3, 5]. In this patient, the infected aortic aneurysm was successfully treated by debridement and omental filling, although we could not prepare a rifampicin-soaked graft owing to an emergency case.

Revascularization for the right lower limb was initially considered. We assumed bypass for the lower extremities as an option, but it was not performed because the patient complained of only mild claudication and had no trouble performing his daily activities [4]. Because femoral arteries were exposed for partial CPB and the patient required revascularization sooner or later, we should have grafted the bilateral femoral arteries in the first surgery.

Reports of intestinal reconstruction vary from case to case [7, 8]. Intestinal one-stage reconstruction to connect the duodenum and jejunum during the first procedure is simpler and easier than two-stage reconstruction; however, a minor leak following intestinal reconstruction can result in reinfection. We chose a two-stage reconstruction with initial resection of the fistula and an enterostomy for feeding, and a subsequent duodenojejunostomy after recovery, to ensure safety. Although the two-stage reconstruction requires a longer hospital stay than one-stage reconstruction, we believe it is more beneficial for patients in terms of decreasing morbidity and mortality in such unstable condition than one-stage surgery.

There is a room for further consideration about the order of procedures of an intestinal resection and revascularization by graft replacement. In the present case, revascularization preceded the intestinal resection similar to previous studies [9–11]. The patient actually recovered well. However, grafting in the irrigated surgical field after the intestinal resection may be an ideal order of procedure for decreasing the amount of bacteria around the graft. This order remains a debatable issue to be discussed with the digestive surgeons.

In conclusion, we reported on an extremely rare occurrence of an infected thoracoabdominal aneurysm associated with Leriche syndrome, which was successfully treated with an in situ four-branched vessel graft replacement, omental filling, and two-stage intestinal reconstruction. The prompt diagnosis and aggressive surgical treatment saved the patient with an infected aortic aneurysm and aortoduodenal fistula.

## References

[1] Tabuchi A., Masaki H., Yunoki Y. (2007). Late Outcome of Extra-anatomic Bypass for Infected Abdominal Aortic Aneurysm. *Japanese Journal of Vascular Surgery*.

[2] Yasuda T., Yamamoto S., Ishida Y. (1999). A case of impending rupture of an infected thoracoabdominal aortic aneurysm. *Japanese Journal of Cardiovascular Surgery*.

[3] Hsu R. B., Chang C. I., Chan C. Y., Wu I. H. (2011). Infected aneurysms of the suprarenal abdominal aorta. *Journal of Vascular Surgery*.

[4] Azizi F., Reichman B. L., De Groot H. G., Van der Laan L. (2012). Primary aortoduodenal fistula in combination with aortoiliac occlusive disease: report of a rare case. *Journal of Cardiovascular Surgery*.

[5] Ting A. C. W., Cheng S. W. K., Ho P., Poon J. T. C., Tsu J. H. L. (2005). Surgical treatment of infected aneurysms and pseudoaneurysms of the thoracic and abdominal aorta. *American Journal of Surgery*.

[6] Kim T. H., Ko Y. G., Kim U. (2011). Outcomes of endovascular treatment of chronic total occlusion of the infrarenal aorta. *Journal of Vascular Surgery*.

[7] dos Santos C. R., Casaca R., de Almeida J. C. M., Mendes-Pedro L. (2014). Enteric repair in aortoduodenal fistulas: a forgotten but often lethal player. *Annals of Vascular Surgery*.

[8] Cendan J. C., Thomas JB 4th, Seeger J. M. (2004). Twenty-one cases of aortoenteric fistula: lessons for the general surgeon. *The American Surgeon*.

[9] Ho S., Liu B., Loya R., Koury I. (2016). Primary aortoenteric fistula: a rare case of a massive gastrointestinal bleed. *Cureus*.

[10] Varetto G., Gibello L., Trevisan A., Castagno C., Garneri P., Rispoli P. (2015). Primary aortoenteric fistula of a saccular aneurysm: case study and literature review. *Korean Circulation Journal*.

[11] Philippakis G. E., Moustardas M. (2013). Surgical treatment of primary aortojejunal fistula. *International Journal of Surgery Case Reports*.

